# Association of dietary patterns with the risk of central obesity in Chinese adults: the China Nutrition and Health Surveillance 2015–2017

**DOI:** 10.3389/fnut.2025.1579434

**Published:** 2025-05-29

**Authors:** Jing Nan, Yuxiang Yang, Fusheng Li, Shuya Cai, Wei Piao, Liyun Zhao, Jiao Xu, Dongmei Yu

**Affiliations:** ^1^National Institute for Nutrition and Health, Chinese Center for Disease Control and Prevention, Beijing, China; ^2^NHC Key Laboratory of Public Nutrition and Health, Chinese Center for Disease Control and Prevention, Beijing, China; ^3^China National Center for Food Safety Risk Assessment, Beijing, China

**Keywords:** dietary pattern, central obesity, factor analysis, cross-sectional studies, China

## Abstract

**Background:**

Diet is one of the important factors affecting obesity, especially central obesity. Dietary patterns can reflect the comprehensive effect of food and nutrients more comprehensively and truthfully, and effectively study the relationship between diet and human health. Therefore, it is of great significance to study the association between dietary patterns and central obesity to promote the change of residents' dietary behavior in the direction of healthier.

**Objectives:**

The purpose of this study was to extract the dietary patterns among Chinese adults aged 18 and above and to explore its relationship with central obesity.

**Method:**

The data was derived from the China Nutrition and Health Surveillance (2015–2017), and the sample participants were obtained by stratified, multi-stage, and random sampling. A total of 61,222 adults aged 18 or older with complete dietary information from mainland China were included in this study. The dietary frequency questionnaire was used to collect food intake information of the respondents, exploratory factor analysis was used to analyze the dietary patterns, and the relationship between dietary patterns and central obesity was analyzed by multivariate logistic regression.

**Results:**

Four dietary patterns were extracted: classical pattern, vegetarian pattern, sugar-oil pattern, and western staple food pattern. The analysis showed that the higher the adherence to the classical pattern, the lower the risk of central obesity. In contrast, higher adherence to the sugar-oil pattern was associated with a higher risk of central obesity.

**Conclusion:**

The risk of central obesity was lower in people with classic pattern. The sugar-oil pattern was not conducive to reducing the risk of central obesity in Chinese adults. These findings provide nationally representative evidence for the development of strategies for the prevention and control of central obesity in Chinese adults.

## 1 Introduction

Obesity is classified as central or peripheral based on fat distribution (1). Fat mainly accumulates in the abdomen is characteristic of central obesity (2). Related studies have shown that compared with peripheral obesity, individuals with this type of obesity have a stronger correlation with multiple chronic diseases (3). With economic development and dietary shifts, central obesity prevalence has risen globally (4). In the epidemiological investigation of nearly 20 years at home and abroad, the average waist circumference and the incidence of central obesity were on the rise (5–7). Diet is a key determinant of obesity, particularly central obesity (8–10).

Traditional nutritional epidemiology focuses on single foods/nutrients, but their individual impacts on chronic diseases are marginal (11, 12). Dietary pattern can more comprehensively and realistically reflect the comprehensive effects of food and nutrients, and also help effectively investigate the relationship between diet and human health (13). While China has some dietary pattern studies, nationally representative research on central obesity-dietary pattern associations remains limited (14, 15).

Therefore, it is necessary to study the association between dietary patterns and central obesity, which is of great significance to promote the change of residents' dietary behaviors in a healthier direction. This study aimed to identify dietary patterns in Chinese adults using nationally representative data and evaluate their association with central obesity, to provide a basis for the development and the improvement of nutritional dietary guidance related to central obesity.

## 2 Materials and methods

### 2.1 Study design and participants

#### 2.1.1 Study design

The data was from the China Nutrition and Health Surveillance (2015–2017). The target population was adults in 2015. The sampling design used a stratified, multi-stage, and random sampling method to extract representative samples from 31 provinces/autonomous regions/municipalities in mainland China in 2015. Considering the distribution balance of stratified factors such as region and the existing work foundation and conditions, 298 survey sates were finally investigated. More detailed information about this survey could be found in our previous report (16). The project was approved by the Ethics Review Committee of Chinese Center for Disease Control and Prevention (approval number: 201519-B). All the participants had signed informed consent before the investigation.

#### 2.1.2 Inclusion and exclusion criteria for the participants

The inclusion and exclusion criteria were as follows: (1) participants aged 18 years or older; (2) the basic information survey, physical examination, dietary interview of the content was completed.

The exclusion criteria were as follows:(1) pregnant women were not included; (2) those whose basic information (gender, age, education level, living areas, income and so on)did not conform to logic were not included; (3) those with missing or abnormal medical examination data: waist circumference ≤ 45 cm or ≥150 cm, height ≤ 100 cm or ≥200 cm, weight ≤ 30 kg or ≥150 kg; (4) those with missing weight data; (5) exclude cases with duplicate information; (6) those with missing or illogical dietary information; (7) those with abnormal energy intake: define the 5th and 95th percentiles of adult energy intake as abnormal energy intake, that is, those with <500 kcal/d or >5,000 kcal/d; (8) those with missing other covariates data.

The current study was finally included 61,222 participants (Table 1). The result was nationally representative.

**Table 1:** General characteristics of participants stratified by gender group in CNHS 2015–2017.

**Variable**	Male (*N*, %)^*^	Female (*N*, %)^*^	Total (*N*, %)^*^
*N*	28,799 (47.04)	32,423 (52.96)	61,222 (100)
**Age** ^#^			
18–29	2,318 (8.05)	2,846 (8.78)	5,164 (8.43)
30–39	3,263 (11.33)	4,083 (12.59)	7,346 (12.00)
40–49	6,200 (21.53)	7,470 (23.04)	13,670 (22.33)
50–59	7,075 (24.57)	8,028 (24.76)	15,103 (24.67)
60–69	6,714 (23.31)	7,073 (21.81)	13,787 (22.52)
≥70	3,229 (11.21)	2,923 (9.02)	6,152 (10.05)
**Education level** ^#^			
Low	11,182 (38.83)	17,625 (54.36)	28,807 (47.05)
Moderate	15,166 (52.66)	12,412 (38.28)	27,578 (45.05)
High	2,451 (8.51)	2,386 (7.36)	4,837 (7.90)
**Marital status** ^#^			
Unmarried	1,507 (5.23)	850 (2.62)	2,357 (3.85)
Married	26,498 (92.01)	29,761 (91.79)	56,259 (91.89)
Divorced/ widowed	794 (2.76)	1,812 (5.59)	2,606 (4.26)
**Living area** ^#^			
Urban	11,852 (41.15)	13,858 (42.74)	25,710 (41.99)
Rural	16,947 (58.85)	18,565 (57.26)	35,512 (58.01)
**Economic regions**			
Eastern	11,083 (38.48)	12,516 (38.60)	23,599 (38.55)
Central	8,448 (29.33)	9,346 (28.83)	17,794 (29.06)
Western	9,268 (32.18)	10,561 (32.57)	19,829 (32.39)
**Region of China**			
Northern	4,277 (14.85)	4,837 (14.92)	9,114 (14.89)
Northeast	2,941 (10.21)	3,237 (9.98)	6,178 (10.09)
Eastern	7,903 (27.44)	8,708 (26.86)	16,611 (27.13)
Central	3,619 (12.57)	4,122 (12.71)	7,741 (12.64)
Southwest	3,758 (13.05)	4,618 (14.24)	8,376 (13.68)
Northwest	3,917 (13.60)	4,143 (12.78)	8,060 (13.17)
Southern	2,384 (8.28)	2,758 (8.51)	5,142 (8.40)
**Average annual household income** ^#^			
Low	6,269 (21.77)	6,969 (21.49)	13,238 (21.62)
Moderate	6,631 (23.03)	7,534 (23.24)	14,165 (23.14)
High	8,258 (28.67)	9,406 (29.01)	17,664 (28.85)
Very high	7,641 (26.53)	8,514 (26.26)	16,155 (26.39)
**Smoking status** ^#^			
Never	9,987 (34.68)	31,269 (96.44)	41,256 (67.39)
Former	14,697 (51.03)	894 (2.76)	15,591 (25.47)
Current	4,115 (14.29)	260 (0.80)	4,375 (7.15)
**Physical activity** ^#^			
Insufficient	3,429 (11.91)	2,996 (9.24)	6,425 (10.49)
Sufficient	25,370 (88.09)	29,427 (90.76)	54,797 (89.51)
**Sleep duration** ^#^			
≤ 6 h	5,054 (17.55)	5,791 (17.86)	10,845 (17.71)
7–8 h	17,578 (61.04)	19,177 (59.15)	36,755 (60.04)
≥9 h	6,167 (21.41)	7,455 (22.99)	13,622 (22.25)
**Screen time** ^#^			
≤ 5 h	24,974 (86.72)	29,023 (89.51)	53,997 (88.20)
>5 h	3,825 (13.28)	3,400 (10.49)	7,225 (11.80)
**BMI** ^#^			
Underweight	942 (3.27)	1,261 (3.89)	2,203 (3.60)
Normal	13,185 (45.78)	14,911 (45.99)	28,096 (45.89)
Overweight	10,613 (36.85)	11,278 (34.78)	21,891 (35.76)
Obese	4,059 (14.09)	4,973 (15.34)	9,032 (14.75)

^*^The data outside the brackets indicates the number of subjects, and the data inside the brackets indicates the composition ratio (%). BMI, body mass index. Due to rounding, the value of multi-category variables may not reach 100%. ^#^Indicated *P*-value <0.05.

### 2.2 Survey content

The China Nutrition and Health Surveillance (2015–2017) collected data from four parts: basic information survey, physical examination, dietary interview, and laboratory test. This study used the information obtained from basic information survey, physical examination, and dietary interview.

To ensure the quality of data, the national quality control working groups and the provincial quality control working groups was set up in the China Nutrition and Health Surveillance (2015–2017). The quality control was carried out in strict accordance with the principles of a unified scheme, manual and questionnaire, unified training and assessment, unified equipment and reagents, and unified data entry and cleaning. The electronic collection method was used throughout the survey to ensure the authenticity and plasticity of the data. After the completion of the survey, the staffs were required to conduct a comprehensive self-examination of the questionnaire, and the quality control staffs of each provincial center for disease control and prevention and monitoring locations were reviewed according to a certain proportion.

#### 2.2.1 Basic information survey

The standard questionnaires were designed by the national project group to collect information. The questionnaire includes: community, family, individual. In addition to the community questionnaire, the information of the sample participants was collected face-to-face by the trained CDC staffs. The basic information questionnaire included age, gender, education level, living areas, income and so on.

#### 2.2.2 Physical examination

Physical examination included the measurement of height, weight, waist circumference, and other indicators. This survey mainly used the data of waist circumference measurement. For waist circumference, we used the same brand and the same type of waist circumference ruler, with a length of 1.5 m, a width of 1 cm, a minimum scale of 0.1 cm, and an accuracy of 0.1 cm. When measuring waist circumference, the participants were asked to be on an empty stomach, upright, relaxed in the abdomen, and the arms were naturally drooped on both sides of the body. The waist measurement needed to be repeated twice to ensure that each measurement error was <2 cm.

#### 2.2.3 Dietary interview

Dietary information was assessed using the Food Frequency Questionnaire (FFQ), and the dietary habits of the respondents in the past year were collected by trained staff through face-to-face surveys. According to the FFQ, the investigators asked the respondents about their food intake in the past year filled in the number of times the food was consumed and the corresponding frequency, and calculated the average amount of food consumed each time. The 64 food items of the questionnaire included rice, wheat, vegetables, fruits, soybean and its products, milk and its products, meat, eggs and other categories.

### 2.3 Establishment of dietary pattern

According to the China Food Composition Table (2009) (17), China Food Composition (2018) (18) and the actual food intake of adult, the 64 food items in the FFQ were merged into 27 food groups, including rice (mainly rice and its products), wheat (mainly wheat and its products), other cereals (mainly corn and its products, other cereal products), mixed beans (red beans, mung beans, etc.), tubers (mainly potatoes and sweet potatoes), fried staple food (mainly fried dough sticks, instant noodles, etc.), soybeans and soy products, fresh vegetable, dried vegetable, fungi and algae (mainly mushrooms, kelp, etc.), fresh fruits, dried fruit, milk and dairy products, pork, other meat (mainly beef, mutton, etc.), poultry, entrails, processed meat (mainly lunch meat, sausage, etc.), aquatic product, egg and egg products, western staple food (mainly bread, pastries, biscuits, etc.), nuts (mainly peanuts, melon seeds, walnuts, hazelnuts, etc.), snack (mainly puffed food, ice cream, candy, etc.), sugared beverage, coffee, fruit and vegetable juice, alcohol. Exploratory factor analysis was used to extract dietary patterns (DP).

Principal component analysis and maximum variance rotation were selected. Due to the significant differences in regional characteristics, lifestyle habits, economic levels, and other aspects among the national population, dietary data are complex and diverse. PCA does not rely on specific assumptions about data distribution, shows strong adaptability to various data patterns, can effectively process sample data with different characteristics, and accurately extract representative dietary patterns. The dietary pattern scores were automatically generated by the SAS system. The choice of factors depended on the eigenvalue and the interpretability of the factor. Each DP was named by the characteristics of food variables with an absolute factor loading >0.3. In the DPs, the higher the factor loading, the stronger the positive correlation between the food groups and the dietary patterns, and the higher the consumption of the food. On the contrary, factor loadings with negative values indicated low consumption. In addition, the current study also calculated the DP score of each DP for each participant. A high score indicated that the diet was more compliant with the corresponding DP. All sample participants were divided into four groups according to the quartile of each DP score, and the DP with the highest score was determined to be its representative DP.

### 2.4 Definition of central obesity

The Criteria of Weight for Adults promulgated by China (WS/T 428—2013) points out that the central obesity of Chinese adults can be judged according to the waist circumference (19). Male waist circumference ≥90 cm and female waist circumference ≥85 cm can be determined as central obesity.

### 2.5 Confounding factors

The variables used for multiple adjustments in logistic regression analysis were as follows: (1) Gender included male and female. (2) All adults were divided into 6 groups according to age: 18–29 years old, 30–39 years old, 40–49 years old, 50–59 years old, 60–69 years old, and 70 years old or over. (3) Marital status included unmarried, married, and divorced/widowed. (4) Education level was divided into low (primary school or below), moderate (junior school), and high (high school or above). (5) Living areas included urban and rural areas. (6) The economic regions were divided into eastern, central, and western regions. (7) The region was divided into Northern China, Northeast China, Eastern China, Central China, Southwest China, Northwest China, and Southern China according to geographical location. (8) Income levels were classified as low (<$685), moderate ($685–1,370), high ($1,370–2,603), and very high (≥$2,603). (9) Smoking status was categorized as never smoked, formerly smoked, or currently smoked. (10) Physical activity status was decided based on the total weekly duration of different exercise levels: The total time of moderate-intensity activity within 1 week was <150 min, high-intensity activity was <75 min, or the cumulative amount of moderate and high-intensity activity was <150 min, which was defined as insufficient physical activity, otherwise, it was defined as sufficient activity. (11) Sleep duration was categorized as <6 hours, 7–8 h, or over 9 h per day. (12) Screen time of more than 5 h per day was defined as excessive screen time, no more than the screen time was normal. (13) Body mass index (BMI) (19) was classified as underweight (BMI <18.5 kg/m^2^), normal (18.5 ≤ BMI <24 kg/m^2^), overweight (24 ≤ BMI <28 kg/m^2^), and obese (BMI ≥ 28 kg/m^2^) according to The Criteria of Weight for Adults promulgated by China (WS/T 428—2013).

### 2.6 Statistical analysis

**Figure 1:**
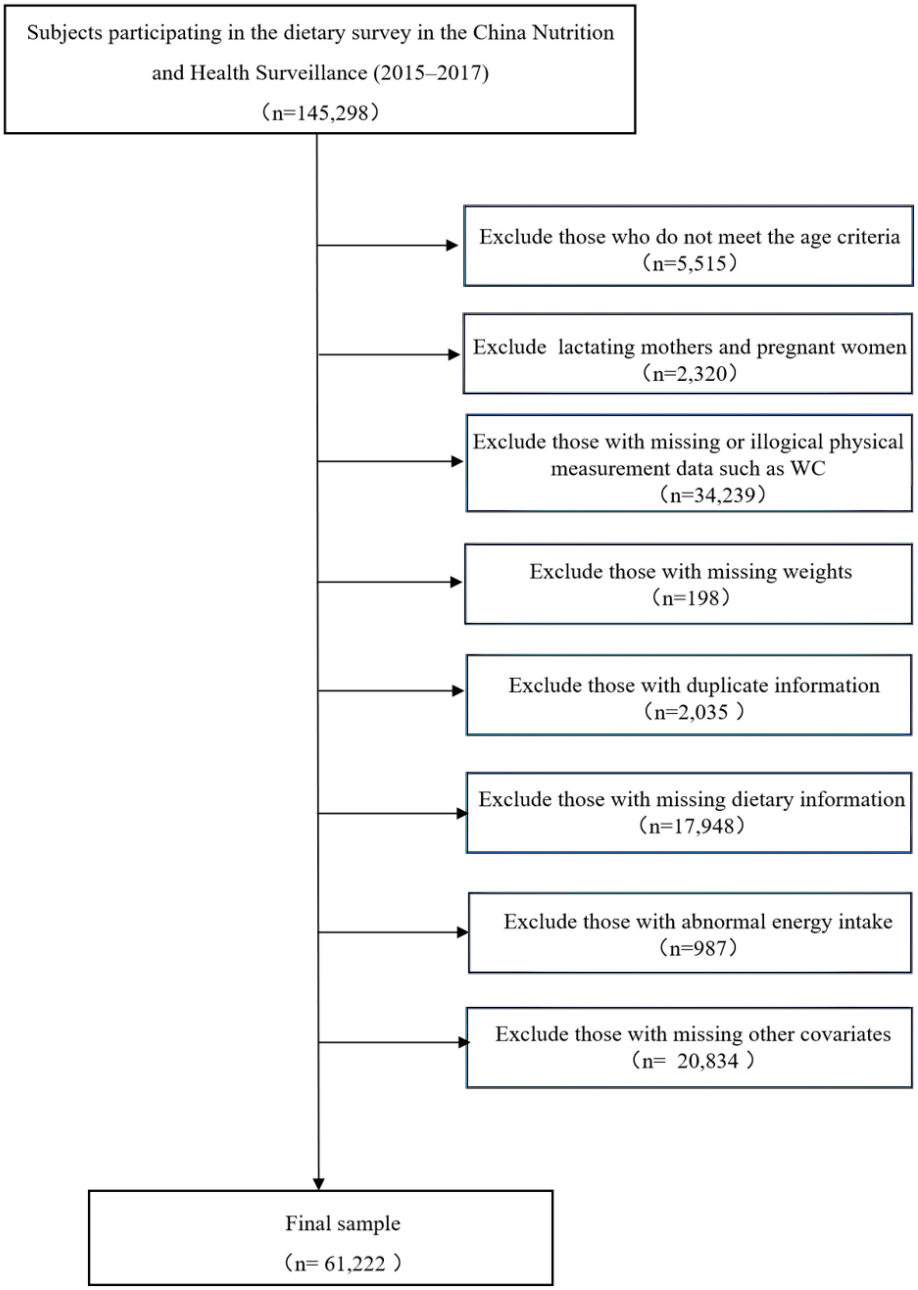
Flowchart of research sample screening process.

SAS 9.4 software (SAS Institute Inc., Cary, NC, USA) was used to clean and analyze all statistical data, and R 4.1.0 software was used for drawing in this study. The categorical variables of the sample's baseline characteristics were represented by the number and percentage. The rate was calculated by the complex sampling weighting method. The basic sampling weight was calculated according to the sampling design, and the post-stratification weight was obtained through the data from the China National Bureau Statistics in 2010. The final weight was the product of the basic sampling weight and the post-stratification weight. DPs were extracted by exploratory factor analysis. To maintain the national representativeness of the data, the PROC SURVEYFREQ was used to calculate the rate. Factor analysis was conducted by PROC FACTOR. The relationship between DPs and central obesity and its components was analyzed by multivariate logistic regression and the models were adjusted by potential confounding covariates. Model 1 did not make any adjustments. Model 2 was adjusted for age, gender, education level, marital status, urban and rural areas, regions, and income. Model 3 was further adjusted for smoking status, BMI, physical activity, sleep duration, and screen time. Logistic regression took the occurrence of central obesity as the dependent variable and dietary pattern as the independent variable, and it took the Q1 group as the reference group, calculated the odds ratio and 95 % confidence interval of the risk of central obesity in other groups, and calculated the *p*-trend in different dietary patterns. The chi-square test checked the statistical difference of categorical variables among the groups. A two-sided *p* < 0.05 was defined as statistical significance. The screening process of research samples is shown in Figure 1.

## 3 Results

### 3.1 General characteristics of the participants

A total of 61,222 adults aged 18 or over were included in this study, 28,799 males and 32,423 females, 25,710 from urban areas and 35,512 from rural areas (Table 1). The average age of males was slightly higher than that of females (52.80 vs. 51.60). The average BMI of the subjects was (24.30 ± 3.61) kg/m^2^, and the mean value of women was slightly higher than that of men (24.32 vs. 24.27). There were significant differences in the living area (urban or rural), education level, marital status, smoking status, physical activity, screen time, and sleep duration between different genders (*p* < 0.05).

### 3.2 Dietary patterns and its distribution among Chinese adults

**Figure 2:**
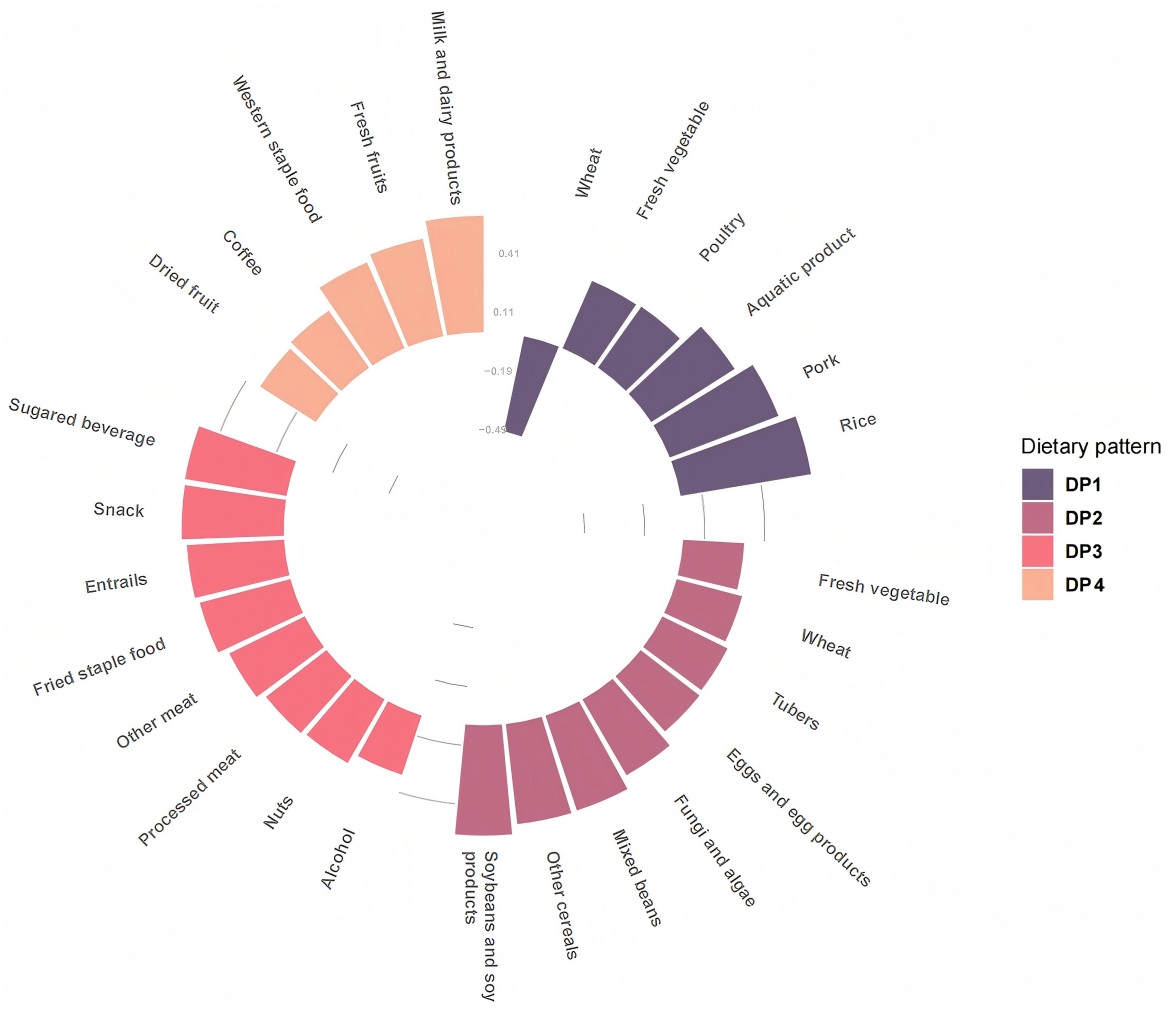
Factor loading of food items in each dietary pattern.

Based on the actual food intake of the subjects, the dietary pattern was extracted by exploratory factor analysis, and the Kaiser–Meyer–Olkin value (KMO) was 0.732, suggesting that there was a strong correlation between various food groups. The result of Barlett's sphericity test was statistically significant (*p* < 0.001). The results of the KMO test and Barlett's sphericity test suggested that the dietary data of the subjects were suitable for exploratory factor analysis. Therefore, the daily intake of each food group of the subjects was included in the model for exploratory factor analysis. Based on the eigenvalue ≥0.3, combined with the slope of the gravel map and the interpretability of the dietary pattern, four dietary patterns were finally extracted, which explained 26.92 % of the variance of 27 food groups. The four dietary patterns could explain 10.03 %, 6.95%, 5.21 %, and 4.73 % of the variation, respectively. DP1 was named as “classical pattern”(rich in rice, pork, aquatic products, poultry, and fresh vegetables, but low in wheat), DP2 was named as “vegetarian pattern”, characterized by high factors loading from soybeans and soy products, other cereals, mixed beans, fungi and algae, eggs and egg products, tubers, wheat. Similarly, the other two DPs were named DP3sugar-oil pattern (rich in sugary beverage, snack, entrails, fried staple food, other meat, processed meat, and nuts), DP4—western staple food pattern (rich in milk and dairy products, fresh fruit, western staple food, coffee, and dried fruit), as shown in Figure 2.

The distribution results of the four DPs are shown in Table 2. The proportion of DP1–DP4 in adult residents was 33.90%, 26.41%, 18.16%, and 21.53%, respectively. DP1 (classical pattern) was the main DP of all subjects. The DP of Northeast and Southwest was mainly DP2 (vegetarian pattern), and that of other regions was DP1. DP4 was more common in the high-income group. The dietary pattern of the screen time >5 h group was also DP4. Overall, the differences between the four dietary pat-terns were statistically significant. Among the groups with different characteristics, except for the BMI group, the differences in dietary patterns among the other groups were statistically significant (*p* < 0.05).

**Table 2:** Characteristics of 4 dietary patterns.

**Variable**	DP1 (*N*, %)^*^	DP2 (*N*, %)^*^	DP3 (*N*, %)^*^	DP4 (*N*, %)^*^	***P*-value**
All	22,071 (33.90)	16,908 (26.41)	10,097 (18.16)	12,146 (21.53)	<0.0001
**Sex**					<0.0001
Male	10,931 (36.15)	7,442 (24.60)	5,020 (20.48)	5,406 (18.77)	
Female	11,140 (31.53)	9,466 (28.31)	5,077 (15.71)	6,740 (24.45)	
**Age**					<0.0001
18–29	1,577 (27.63)	1,252 (23.43)	1,214 (24.88)	1,121 (24.06)	
30–39	2,536 (33.48)	1,830 (25.21)	1,338 (17.09)	1,642 (24.22)	
40–49	5,331 (37.86)	3,700 (27.29)	2,227 (16.29)	2,412 (18.56)	
50–59	5,868 (36.95)	4,059 (26.82)	2,312 (16.04)	2,864 (20.19)	
60–69	4,982 (36.77)	3,999 (29.00)	2,078 (15.14)	2,728 (19.09)	
≥70	1,777 (31.72)	2,068 (31.97)	928 (14.75)	1,379 (21.56)	
**Education level**					<0.0001
Low	11,398 (38.99)	8,644 (30.46)	4,413 (15.36)	4,352 (15.19)	
Moderate	9,648 (34.07)	7,222 (25.66)	4,882 (19.91)	5,826 (20.37)	
High	1,025 (22.39)	1,042 (20.14)	802 (18.44)	1,968 (39.04)	
**Marital status**					<0.0001
Unmarried	642 (22.31)	568 (23.34)	561 (27.39)	586 (26.95)	
Married	20,574 (35.49)	15,502 (26.69)	9,157 (17.01)	11,026 (20.81)	
Divorced/Widowed	855 (33.36)	838 (29.74)	379 (15.55)	534 (21.36)	
**Living area**					<0.0001
Urban	7,434 (28.71)	6,882 (24.82)	4,484 (19.27)	6,910 (27.21)	
Rural	14,637 (39.63)	10,026 (28.16)	5,613 (16.94)	5,236 (15.28)	
**Economic regions**					0.0009
Eastern	7,959 (32.79)	6,315 (23.48)	3,948 (18.55)	5,377 (25.18)	
Central	5,778 (33.46)	5,546 (28.95)	3,121 (18.53)	3,349 (19.05)	
Western	8,334 (36.35)	5,047 (28.19)	3,028 (17.03)	3,420 (18.44)	
**Region of China**					<0.0001
Northern	3,439 (34.47)	2,065 (22.93)	1,411 (16.72)	2,199 (25.88)	
Northeast	1,263 (16.54)	1,808 (29.18)	1,409 (25.94)	1,698 (28.34)	
Eastern	5,329 (32.29)	4,895 (25.92)	2,846 (17.45)	3,541 (24.34)	
Central	2,639 (38.44)	2,456 (28.02)	1,392 (18.75)	1,254 (14.79)	
Southwest	2,370 (26.00)	2,952 (35.42)	1,493 (18.64)	1,561 (19.94)	
Northwest	4,520 (52.29)	1,172 (16.03)	1,111 (15.64)	1,257 (16.03)	
Southern	2,511 (45.56)	1,560 (22.68)	435 (14.70)	636 (17.07)	
**Average annual household income**					<0.0001
Low	5,651 (40.90)	3,881 (29.26)	1,887 (15.43)	1,819 (14.41)	
Moderate	5,833 (38.19)	4,056 (29.48)	2,210 (17.40)	2,066 (14.92)	
High	6,236 (33.28)	4,905 (26.50)	3,170 (19.86)	3,353 (20.36)	
Very high	4,351 (26.86)	4,066 (22.21)	2,830 (18.76)	4,908 (32.17)	
**Smoking status**					<0.0001
Never	14,525 (32.55)	11,781 (27.22)	6,566 (16.66)	8,384 (23.56)	
Former	5,928 (36.74)	3,962 (24.29)	2,808 (21.96)	2,893 (17.02)	
Current	1,618 (36.07)	1,165 (26.98)	723 (17.56)	869 (19.39)	
**Physical activity**					0.0001
Insufficient	2,458 (35.73)	1,889 (28.76)	1,057 (18.69)	1,021 (16.83)	
Sufficient	19,613 (33.67)	15,019 (26.11)	9,040 (18.09)	11,125 (22.12)	
**Sleep duration**					0.0263
≤ 6 h	3,747 (33.95)	3,098 (27.83)	1,781 (17.98)	2,219 (20.24)	
7–8 h	13,114 (33.14)	10,002 (26.04)	6,130 (18.50)	7,509 (22.33)	
≥9 h	5,210 (36.09)	3,808 (26.55)	2,186 (17.30)	2,418 (20.06)	
**Screen time**					<0.0001
≤ 5 h	20,119 (35.86)	15,146 (27.24)	8,633 (16.84)	10,099 (20.06)	
>5 h	1,952 (25.82)	1,762 (22.98)	1,464 (23.60)	2,047 (27.60)	
**BMI**					0.3122
Underweight	782 (36.12)	712 (27.22)	357 (17.84)	352 (18.82)	
Normal	10,117 (32.65)	7,941 (26.65)	4,444 (17.91)	5,594 (22.79)	
Overweight	7,951 (35.14)	5,861 (25.79)	3,684 (18.20)	4,395 (20.87)	
Obese	3,221 (34.40)	2,394 (26.79)	1,612 (18.97)	1,805 (19.84)	

^*^The data outside the brackets indicates the number of subjects, and the data inside the brackets indicates the composition ratio (%). BMI, body mass index. Due to rounding, the value of multi-category variables may not reach 100%. DP1 was named as “classical pattern”, DP2 was named as “vegetarian pattern”, DP3 was named as “sugar-oil pattern”, DP4 was named as “western staple food pattern”.

### 3.3 Associations between dietary patterns and central obesity

The association between dietary patterns and central obesity is shown in Table 3. Adults with higher scores for the classical pattern had lower prevalence rates of central obesity. In contrast, central obesity was more common in adults with higher scores for the vegetarian pattern and the western staple food pattern, with central obesity rates as high as 30.63 % and 28.03 % in the Q4 group, respectively.

**Table 3:** Logistic regression of dietary patterns and central obesity.

**Dietary pattern**	**Group of quartile**	**Total**	***N* (%)**	**Model 1**	**Model 2**	**Model 3**
				**OR (95%CI)**	**OR (95%CI)**	**OR (95%CI)**
Classical Pattern	Q1	15,443	5,824 (28.46)	Ref	Ref	Ref
	Q2	15,787	5,808 (28.38)	0.961 (0.918–1.006)	0.921 (0.876–0.968)	0.954 (0.894–1.019)
	Q3	15,279	4,630 (22.62)	0.718 (0.685–0.753)	0.756 (0.715–0.798)	0.86 (0.801–0.924)
	Q4	14,713	4,203 (20.54)	0.660 (0.629–0.693)	0.772 (0.728–0.818)	0.865 (0.802–0.932)
	*P* for trend	–	–	<0.0001	<0.0001	<0.0001
Vegetarian Pattern	Q1	13,866	3,815 (18.64)	Ref	Ref	Ref
	Q2	15,203	4,775 (23.33)	1.206 (1.147–1.269)	1.086 (1.031–1.144)	0.963 (0.963–1.030)
	Q3	15,920	5,607 (27.40)	1.432 (1.363–1.505)	1.199 (1.138–1.263)	1.002 (0.937–1.072)
	Q4	16,233	6,268 (30.63)	1.657 (1.578–1.740)	1.308 (1.241–1.378)	1.042 (0.973–1.115)
	*P* for trend	–	–	<0.0001	<0.0001	0.0959
Sugar-oil Pattern	Q1	15,283	5,229 (25.55)	Ref	Ref	Ref
	Q2	14,927	4,831 (23.61)	0.92 (0.877–0.965)	0.992 (0.945–1.042)	0.941 (0.883–1.002)
	Q3	15,421	5,061 (24.73)	0.939 (0.896–0.985)	1.068 (1.017–1.123)	0.977 (0.916–1.042)
	Q4	15,591	5,344 (26.11)	1.003 (0.957–1.051)	1.267 (1.203–1.335)	1.168 (1.091–1.250)
	*P* for trend	–	–	0.6997	<0.0001	<0.0001
Western staple food Pattern	Q1	14,551	4,276 (20.89)	Ref	Ref	Ref
	Q2	14,797	4,833 (23.62)	1.166 (1.109–1.225)	1.121 (1.065–1.179)	1.077 (1.009–1.150)
	Q3	15,618	5,619 (27.46)	1.35 (1.287–1.417)	1.247 (1.185–1.313)	1.103 (1.033–1.178)
	Q4	16,256	5,737 (28.03)	1.311 (1.249–1.375)	1.15 (1.089–1.215)	1.038 (0.967–1.114)
	*P* for trend	–	–	<0.0001	<0.0001	0.232

Model 1: unadjusted model; Model 2: adjusted for age, gender, education level, marital status, urban and rural areas, regions, and income; Model 3: further adjusted for smoking status, BMI, physical activity, sleep duration, and screen time.

Two dietary patterns had a positive correlation with central obesity when the co-variates were not adjusted. They were vegetarian pattern (Q4 vs. Q1, OR = 1.657,95 % CI = 1.578–1.740, *p*-trend <0.0001) and western staple food pattern (Q4 vs. Q1, OR = 1.311,95 % CI = 1.249–1.375, *p*-trend <0.0001). Meanwhile, the study found that the classical pattern (Q4 vs. Q1, OR = 0.660,95 % CI = 0.629–0.693, *p*-trend <0.0001) was negatively correlated with the risk of central obesity, and the sugar-oil pattern was not significantly associated. After adjusting the covariates of general conditions, the classical pattern was still negatively correlated with the risk of central obesity, and the other three dietary patterns were positively correlated with the risk of central obesity. After adjusting all covariates, the classical pattern (Q4 vs. Q1, OR = 0.865,95 % CI = 0.802-0.932, *p*-trend <0.0001) was still a protective factor for central obesity, while the vegetarian pattern (Q4 vs. Q1, OR = 1.042,95 % CI = 0.973–1.115, *p*-trend = 0.0959) was not statistically significant. Sugar-oil pattern (Q4 vs. Q1, OR = 1.168,95 % CI = 1.091–1.250, *p*-trend <0.0001) was a risk factor for central obesity. The risk of central obesity in the Q2 group and Q3 group of western staple food pattern were 1.077 and 1.103, respectively, compared with the Q1 group.

## 4 Discussion

Dietary is a key factor around them affecting the prevalence of central obesity (20, 21). It is of great significance to study the relationship between dietary pattern and central obesity for promoting healthy eating behavior of residents. There are four dietary patterns proposed by current studies in China, including the northern dietary pattern, the southern dietary pattern, the fast-food dietary pattern and the snack dietary pattern. On this basis, the relationship between dietary patterns and kidney disease, diabetes, hyperlipidemia, hypertension and general obesity was discussed. However, there are not enough nationally representative studies on the relationship between central obesity and dietary patterns in adults in China.

Four main dietary patterns were identified in this study, including the classical pattern, vegetarian pattern, sugar-oil pattern and western staple food pattern. The dietary habits of Chinese adults were well described, explaining 26.92% of the variance in dietary intake. After adjusting confounding factors, it was found that the classical pattern was the protective factor for central obesity, and western staple food pattern was the risk factor.

Among the four dietary patterns, the classical pattern was the main dietary pattern, which was similar to the results of a study by Peking University, indicating that the diet of Chinese adults was still dominated by traditional forms (8). This study found that classical pattern has a protective effect on the occurrence and development of central obesity. This may be related to the fact that rice is a low energy-density food (22). This result was consistent with the traditional Southern pattern proposed by a longitudinal study of the population based on the China Health and Nutrition Survey (CHNS), indicating that the traditional rice-rich dietary pattern was associated with less weight gain (8). The classical pattern was rich in ingredients, which was in line with the dietary habits of Chinese residents, and the food was close to the dietary guidelines (23). A review of dietary patterns and obesity in Chinese adults also shows that traditional Chinese dietary patterns have shown consistent benefits in reducing the risk of obesity in both cross-sectional and longitudinal studies (24). A nationally representative study in South Korea also pointed out that the dietary pattern represented by rice was negatively correlated with obesity (25).

The vegetarian pattern was characterized by soybeans, mixed beans, other cereals, fungi and algae, eggs and egg products, tubers, and wheat. When confounding factors were not adjusted, it was found to be positively correlated with central obesity. However, after adjusting for confounding factors such as BMI, physical activity, screen time, and general information, it was no longer associated with central obesity. This dietary pattern has a higher carbohydrate intake, and a high-carbohydrate eating pattern increases the risk of central obesity (26). A large cohort study in South Korea showed no significant correlation between multi-grain dietary pattern (characterized by very high consumption of multi-grain, along with very low consumption of red meat/high-fat red meat, poultry, wheat-based products, processed meats/red meat by-products, and carbonated beverages) and the incidence of central obesity, consistent with the findings of this study (27). However, this result is different from that of studies in other countries (28, 29). A Japanese study showed that the “healthy Japanese” pattern, which was characterized by foods rich in soy, fungi and algae, was inversely associated with waist circumference and central obesity (30). Thus, the relationship between dietary patterns characterized by soy, algae, and wheat intake and central obesity in Asian populations remains controversial. The association between soy, algae and wheat and central obesity deserves further detailed study.

The consumption of sugary drinks and snacks with high energy density and low nutritional value is an important part of the sugar-oil pattern. This behavior can easily lead to excessive accumulation of energy, thereby promoting the occurrence of metabolic diseases such as obesity (21). Francesco Angelico's research is consistent with the results of this study (29). Studies have shown that regular consumption of junk food such as sugary drinks and snacks can lead to weight gain and accelerate insulin resistance, which plays a key role in the development of central obesity and Metabolic syndrome (MetS) (22). In addition, fried food has a high energy density, and regular consumption of it can easily lead to obesity. Studies have shown that fried food is one of the most dangerous foods to cause hyperlipidemia and coronary heart disease (31). Therefore, dietary recommendations should aim to reduce adherence to this dietary pattern.

Western staple food pattern was mainly composed of milk and dairy products, fruits, western staple food, and coffee, which was positively correlated with the occurrence of central obesity. The western staple food pattern was more common in groups with higher education levels and people with very-high-income levels, which may be related to the more westernized living habits of these groups (32). With the development of social culture, China has become one of the most important coffee-consumption countries in the world, and the consumption of coffee by residents has gradually increased (32). The “coffee and sweets” pattern proposed by a Korean study is similar to the main features of the western food staple pattern, and also shows that dairy products, desserts, coffee, etc. are positively correlated with the incidence of central obesity (27). However, previous studies on the relationship between coffee intake and systemic and central obesity have reported conflicting results. A Mendelian randomized study involving two large general population cohorts (20) showed that high coffee intake was associated with lower risk of obesity and metabolic syndrome. Asian residents have different cultures and eating habits than their Western counterparts, and Chinese and Korean residents may consume more instant coffee instead of filtered coffee, resulting in an intake of extra sugar and energy (33). A study found that instant coffee consumers were more likely to develop systemic obesity and central obesity than non-consumers, while no significant association was observed between filtered coffee consumers (34). Milk and dairy products are widely recommended as part of a healthy diet, but milk contains fat and sugar, which if consumed too much can likewise lead to an energy surplus and increase the risk of obesity (35). The abnormal intake of instant coffee and sugary dairy products should attract the attention of relevant national authorities.

The results of this study not only demonstrate the important role of joint analysis of food on central obesity in Chinese adults, but also provide practical recommendations for future nutritional intervention programs, which may have important implications for public health practice. We suggest that increasing the frequency of healthy food intake, insisting on dietary diversity and eating a greater variety of healthy foods may be beneficial to reducing the risk of central obesity.

The main limitation of this study was the cross-sectional nature of the data, which made it impossible to draw a causal relationship between DP and central obesity. Secondly, we used the FFQ questionnaire to assess the dietary intake throughout the year, which may cause recall bias. Thirdly, the factor analysis approach used in this study relied to some extent on current dietary data. Therefore, the cumulative variance contribution rate of this study was not high (26.92 %), and the results obtained were difficult to reproduce in other studies, but at least reflect the dietary status of the population under certain conditions. In addition to its limitations, our research also has merits. First of all, we used the nationally representative data of Chinese adult population to obtain the results, and the results could be extended to the whole population. Furthermore, we considered several covariates when setting the logistic regression model to obtain results as close as possible to reality.

## 5 Conclusions

The current study identified four dietary patterns and explored their association with central obesity. The findings suggest that people who stick to the classical pattern have a lower risk of developing central obesity, while adherence to the sugar-oil pattern was not conducive to reducing the risk of central obesity in Chinese adults. The combination of rice, aquatic products, poultry, fresh vegetables, pork and other foods is beneficial to human health. These products could be beneficial to human health, but as part of a dietary pattern, specifically the Chinese classical dietary pattern. This suggests that effective dietary interventions can be used as an adjunct treatment to improve central obesity in adults. These findings also provide nationally representative evidence for the development of central obesity prevention and control strategies.

## Data Availability

The original contributions presented in the study are included in the article/supplementary material, further inquiries can be directed to the corresponding author/s.
